# RIPK4 promotes bladder urothelial carcinoma cell aggressiveness by upregulating VEGF-A through the NF-κB pathway

**DOI:** 10.1038/s41416-018-0116-8

**Published:** 2018-06-05

**Authors:** Jian-Ye Liu, Qing-Hai Zeng, Pei-Guo Cao, Dan Xie, Xin Chen, Fei Yang, Le-Ye He, Ying-Bo Dai, Jing-Jing Li, Xiao-Ming Liu, Hong-Liang Zeng, Yi-Xin Zhu, Lian Gong, Yan Cheng, Jian-Da Zhou, Jun Hu, Hao Bo, Zhen-Zhou Xu, Ke Cao

**Affiliations:** 1 Department of Urology, The Third Xiangya Hospital of Central South University, 410013 Changsha, China; 2 Institute of Prostate Disease of Central South University, 410013 Changsha, China; 3 Department of Dermatology, The Third Xiangya Hospital of Central South University, 410013 Changsha, China; 4 Department of Onology, The Third Xiangya Hospital of Central South University, 410013 Changsha, China; 5 Department of Pathology, Sun Yat-sen University Cancer Center, 510060 Guangzhou, China; 6 State Key Laboratory of Oncology in South China, 510060 Guangzhou, China; 7 Department of Urology, Sun Yat-sen University Cancer Center, 510060 Guangzhou, China; 8 School of Public Health, Central South University, 410078 Changsha, China; 9 Department of Plastic Surgery, The Third Xiangya Hospital of Central South University, 410013 Changsha, China; 10 Department of Gastroenterology, The Third Xiangya Hospital of Central South University, 410013 Changsha, China; 11 Hunan Key Laboratory of Pharmacodynamics and Safety Evaluation of New Drugs, 410331 Changsha, China; 12 Department of Pharmacology, School of Pharmaceutical Sciences, Central South University, 410008 Changsha, China; 13 Department of Tissue-Bank, Hunan Cancer Hospital, 410006 Changsha, China; 14 Institute of Reproductive and Stem Cell Engineering, Central South University, 410083 Changsha, China; 15 Department of Urology, Hunan Cancer Hospital, 410006 Changsha, China

**Keywords:** Bladder cancer, Tumour biomarkers, Metastasis

## Abstract

**Background:**

Constitutively activated nuclear factor kappa B (NF-κB) signalling plays vital roles in bladder urothelial carcinoma (BC) progression. We investigate the effect of receptor-interacting protein kinase 4 (RIPK4) on NF-κB activation and BC progression.

**Methods:**

The expression of RIPK4 was examined in 25 cryopreserved paired bladder samples and 112 paraffin BC specimens. In vivo and in vitro assays were performed to validate effect of RIPK4 on NF-κB pathway-mediated BC progression.

**Results:**

High expression of RIPK4 was observed in BC tissues and was an independent predictor for poor overall survival. Up or downregulating the expression of RIPK4 enhanced or inhibited, respectively, the migration and invasion of BC cells in vitro and in vivo. Mechanistically, RIPK4 promoted K63-linked polyubiquitination of tumour necrosis factor receptor-associated factor 2 (TRAF2), receptor-interacting protein (RIP) and NF-κB essential modulator (NEMO). RIPK4 also promoted nuclear localisation of NF-κB-p65, and maintained activation of NF-κB substantially, leading to upregulation of VEGF-A, ultimately promoting BC cell aggressiveness.

**Conclusions:**

Our data highlighted the molecular aetiology and clinical significance of RIPK4 in BC: upregulation of RIPK4 contributes to NF-κB activation, and upregulates VEGF-A, and BC progression. Targeting RIPK4 might represent a new therapeutic strategy to improve survival for patients with BC.

## Introduction

Bladder urothelial carcinoma (BC) occupies the first position in terms of incidence and mortality among genitourinary tumours in China.^1^ BC can be classified into non-invasive and invasive subtypes, with the latter posing a greater risk of metastases.^2^ Metastatic BC remains a lethal disease, with few therapeutic choices beyond front-line therapy: patients with metastatic disease have a 5-year survival rate of only 5%.^3,4^ Studies in human bladder tumour specimens and mouse models have implicated multiple signalling pathways in the progression and metastasis of BC.^5,6^ Moreover, some studies have revealed that constitutive activation of nuclear factor kappa B (NF-κB) signalling has a vital role in the progression of BC, and blockade of the NF-κB pathway could suppress angiogenesis and metastasis in BC.^7,8^ Currently, the precise molecular mechanisms of NF-κB pathway regulation in BC are poorly understood.^9^

Receptor-interacting protein kinase 4 (RIPK4) is a member of the RIP kinase family and is a serine/threonine kinase.^10,11^ It was identified initially as an important regulator of keratinocyte differentiation,^12,13^ and its encoding gene is mutated in Bartsocas–Papas syndrome.^14,15^ Recently, some studies indicated that RIPK4 was overexpressed in some types of cancer, such as skin, ovarian, cervical and colorectal cancers,^16,17^ and in a xenograft tumour model, increased RIPK4 expression promoted ovarian cancer.^16^ Additionally, two groups demonstrated that RIPK4 could activate the NF-κB signalling pathway.^18,19^ These observations suggested that RIPK4 is an oncogene, and that the activated NF-κB signalling pathway is involved in the pathogenesis of some malignant diseases. However, the clinical and biological significance of RIPK4 in BC remain largely unknown. Th erefore, extensive investigations on the functions of RIPK4 in BC are required.

This study aimed to evaluate the influence of RIPK4 on NF-κB activation and BC progression. We observed that RIPK4 was upregulated distinctly in BCs and this overexpression correlated significantly with the survival and clinicopathological characteristics of patients with BC. Overexpression of RIPK4 induced, whereas silencing RIPK4 inhibited, the invasion and metastasis of BC in vitro and in vivo. Furthermore, we demonstrated that RIPK4 might have an important function in the control of the aggression and metastasis of BC by promoting K63-linked polyubiquitination of tumour necrosis factor receptor-associated factor 2 (TRAF2), receptor-interacting protein (RIP) and NF-κB essential modulator (NEMO). This results in increased NF-κB activity by facilitating the cytoplasmic–nuclear translocation of NF-κB-p65, ultimately leading to increased vascular endothelial growth factor A (VEGF-A) levels. Our results suggest that RIPK4 is a key player in the invasion and metastasis in BC, and could represent a novel prognostic biomarker and therapeutic target to treat patients with this malignancy.

## Materials and methods

### Ethics statement

This study was conducted according to the ethical standards contained in the Declaration of Helsinki, and in national and international guidelines; the authors’ Institutional Review Board approved the study.

### Cells

These methodologies are described in Supplementary materials and methods.

### Patient information and tissue specimens

One hundred and twelve paraffin-embedded BC specimens were obtained from the Third Xiangya Hospital of Central South University and the Affiliated Cancer Hospital of Xiangya School of Medicine, Central South University from 2004 to 2013. All the patients with BC were diagnosed histopathologically and were treated with radical cystectomy. The criteria of the World Health Organization (WHO) and the 6th edition of the tumour-nodes-metastasis (TNM) classification of the American Joint Committee on Cancer (AJCC) criteria were used to grade and stage the tumours. Prior patient consent and approval from the Institutional Research Ethics Committee were obtained to use of these clinical materials for research purposes. Details of the clinical information concerning the samples are described in Supplementary Table S1. Twenty-five BC specimens and their matched adjacent non-cancerous bladder urothelial tissues were frozen and stored in liquid nitrogen for subsequent experiments.

### Vectors, retroviral infection and transfection

The vector pMSCV/RIPK4, which overexpresses human RIPK4, was constructed by subcloning the PCR-amplified human *RIPK4* coding sequence into vector pMSCV (Clontech). To silence the endogenous *RIPK4*, we cloned four short hairpin RNA (shRNA) oligonucleotides (ShRNA-1: GCCGATGTCATTGACCTGTTC, ShRNA-2: GGTCAACGAGGTGGACTTTGA, ShRNA-3: GGGACACCAGCAAACTGATGA and ShRNA-4: GGCCCACCTTCCAAGAAATTA) into vector pSuper-retro-puro to generate pSuper-retro-RIPK4-ShRNA(s). The target sequences of NF-κB-p65 to construct the lentiviral ShRNAs were as follows: NF-κB-p65 ShRNA-1: CCGGATTGAGGAGAAACGT; NF-κB-p65 ShRNA-2: CCATCAACTATGATGAGTT; NF-κB-p65 ShRNA-3: ATGGATTCATTACAGCTTA; NF-κB-p65 ShRNA-4: CTCTTCTCAAGTGCCTTAA. The target sequences of VEGF-A to construct lentiviral ShRNAs were as follows: VEGF-A ShRNA-1: GCAGATTATGCGGATCAAACC; VEGF-A ShRNA-2: GCGCAAGAAATCCCGGTATAA; VEGF-A ShRNA-3: GCGAGGCAGCTTGAGTTAAAC; VEGF-A ShRNA-4: GCCAGCACATAGGAGAGATGA. Vector construction, lentivirus production and infection were performed according to previous reports.^20,21^ Stable cell lines expressing RIPK4, RIPK4-ShRNA, NF-κB-p65-ShRNA and VEGF-A-ShRNA were selected for 10 days using 0.5 mg/mL puromycin from 48 h after infection.

### RNA isolation and quantitative real-time reverse transcription polymerase chain reaction (qRT-PCR)

These methodologies are described in Supplementary materials and methods.

### Protein extraction and western blotting

These methodologies are described in Supplementary materials and methods.

### Immunohistochemistry (IHC)

These methodologies are described in Supplementary Materials and Methods. Previous scoring criterions were used for evaluation of the epithelial–mesenchymal transition (EMT) markers (E-cadherin, β-catenin, fibronectin and vimentin),^22^ VEGF-A,^23^ p-p65^24^ and CD82^25^ IHC staining.

### Cell migration assay

These methods are provided in Supplementary materials and methods.

### Matrigel invasion assay

These methods are provided in Supplementary materials and methods.

### Human umbilical vein endothelial cells (HUVECs) tube formation assay

These methods are provided in Supplementary materials and methods.

### Immunoflourescence staining

These methods are provided in Supplementary materials and methods.

### Luciferase activity assays

These methods are provided in Supplementary materials and methods.

### Electrophoretic mobility shift assay (EMSA)

These methods are provided in Supplementary materials and methods.

### Lung metastasis model

These methods are provided in Supplementary materials and methods.

### Statistical analysis

The statistical analysis was carried out using the SPSS 17.0 statistical software package. The relationship between RIPK4 expression and the clinicopathological characteristics was assessed using the *χ*^2^ test. The Kaplan–Meier method was used to plot the survival curves, which were compared using the log-rank test. Univariate and multivariate Cox-regression analyses were used to evaluate the survival data. All experiments were performed at least in triplicate. All data are shown as the mean ± the standard deviation (SD). Analysis of variance (ANOVA) was used to conduct significance tests on the data groups, followed by a Student’s *t* test to compare data between the specific groups. A *P* value less than 0.05 was considered statistically significant.

## Results

### RIPK4 is upregulated in BC

**Fig. 1:**
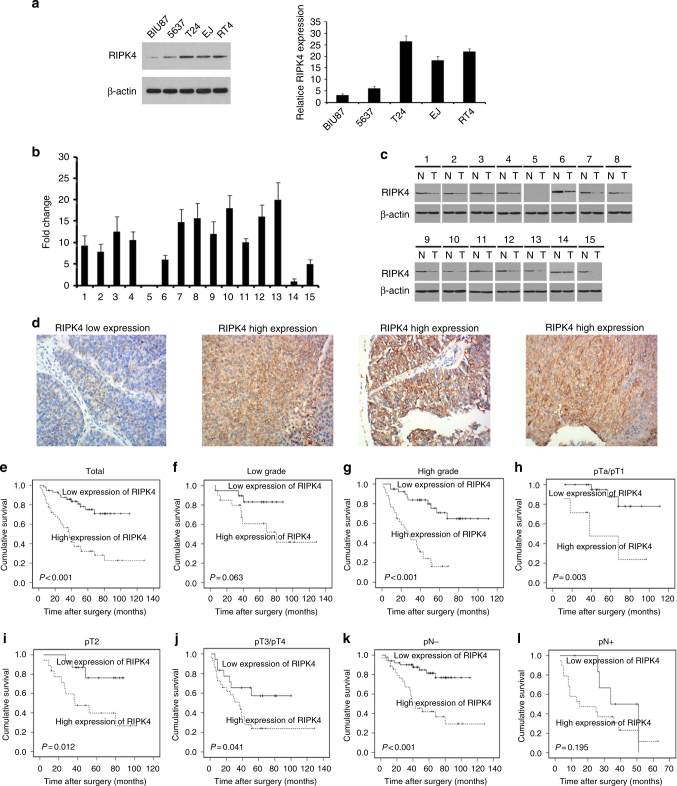
Expression of RIPK4 in BC cell lines and tissues. **a** The levels of the RIPK4 protein in five BC cell lines examined by western blot. **b**, **c** RIPK4 expression increased in BC tissues, compared to the paired adjacent normal bladder urothelial tissues specimens from 15 patients, determined by qRT-PCR analysis and western blot analysis. **d** Representative immunohistochemistry images show the low expression of RIPK4 in one BC tissue sample and high expression of RIPK4 in three BC tissue samples. Original magnification, ×200. **e–l** Kaplan–Meier curves with univariate analyses for patients with low RIPK4 expression versus high RIPK4 expression BC tumours. **e** Kaplan–Meier survival curves comparing cumulative overall survival rates in all patients with BC with low and high RIPK4 expression levels. **f** Survival curves for patients with low-grade disease. **g** Survival curves for patients with high-grade disease. **h** Survival curves for patients with pTa/pT1 disease. **i** Survival curves for patients with pT2 disease. **j** Survival curves for patients with pT3/pT4 disease. **k** Survival curves for patients with pN- disease. **l** Survival curves for patients with pN + disease

To assess the expression pattern of RIPK4 in BC, we analysed the RIPK4 protein level in several BC cell lines, including BIU87, 5637, T24, EJ and RT4 cells, and found that RIPK4 levels were high in T24, EJ and RT4, which show strong invasiveness, and low in BIU87 and 5637, which show weak invasiveness (Fig. 1a). qRT-PCR was performed for 15 pairs of human primary BC tissues and their corresponding non-cancerous bladder urothelial tissues. Overexpression of *RIPK4* at the mRNA level was detected in 86.7% (13/15) of human primary BCs compared with the corresponding non-cancerous tissues (Fig. 1b). Consistent with the results from the qRT-PCR analysis, western blotting showed higher levels of RIPK4 in 86.7% (13/15) of human primary BC tissues compared its level in the matched non-cancerous tissues from the same patients (Fig. 1c). Moreover, the protein level of RIPK4 was detected using IHC in 112 BC clinical tissues that underwent radical cystectomy. The IHC results showed that the RIPK4 protein was present primarily in cytoplasm of cancer cells. High levels of RIPK4 were observed in 54/112 (48.2%) of the BCs, the remaining 58 cases (51.8%) had low levels of RIPK4 (Fig. 1d).

### Association between RIPK4 levels and clinicopathological variables in BCs

Supplementary Table S1 shows the associations between RIPK4 levels and clinicopathological variables in patients with BC. High levels of RIPK4 in BCs were associated significantly with advanced pT status (*P* = 0.003) and pN status (*P* = 0.004). There were no significant associations between the RIPK4 level and other clinicopathological features, including the patients’ age, gender, tumour size, tumour multiplicity or tumour grade.

### The impact of RIPK4 on patient survival

Kaplan–Meier analyses revealed poor overall survival (OS) in patients with BC that showed high levels of RIPK4 (*P* *<* 0.001, Fig. 1e). Furthermore, we found that patients with higher RIPK4 levels survived for a significantly shorter time compared with those with low RIPK4 levels in the high-grade subgroup (*P* < 0.001; Fig. 1g), and in stages pTa/pT1 (*P* = 0.003; Fig. 1h), pT2 (*P* < 0.012; Fig. 1i), pT3/pT4 (*P* = 0.041; Fig. 1j) and pN- (*P* < 0.001; Fig. 1k).

In the univariate analysis, high levels of RIPK4 correlated closely with shorter OS for patients with BC (*P* < 0.001; Supplementary Table S2). Besides the RIPK4 level, the impact value of a patients’ age, patients’ gender, tumour size, tumour multiplicity, tumour grade and stage were also been tested in the univariate analysis for OS. Variables that showed a significant impact on patients’ survival in the univariate analysis are listed in Supplementary Table S2, which were further subjected to multivariate analysis. The results showed that a high RIPK4 level is an independent predictor of poor OS in patients with BC (*P* < 0.001; Supplementary Table S3).

### Downregulation of RIPK4 suppresses BC cell invasion and metastasis in vitro and in vivo

**Fig. 2:**
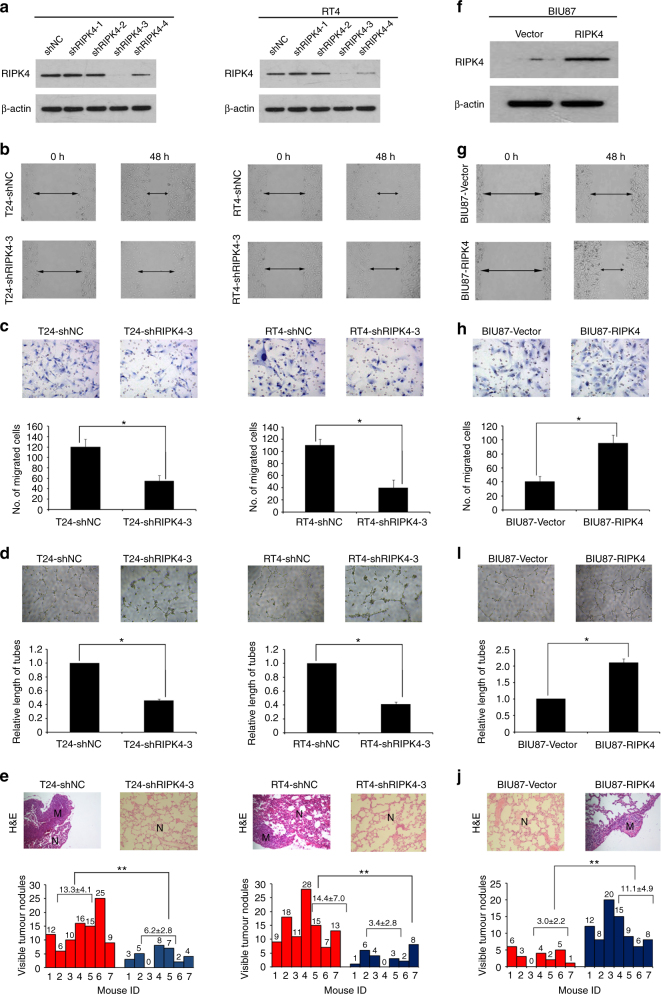
Downregulating or upregulating the expression of RIPK4 enhanced or inhibited, respectively, the migratory and invasive abilities of BC cells in vitro and in vivo. **a** T24 and RT4 cells were infected with lentivirus-expressing *RIPK4* shRNA-1, shRNA-2, shRNA-3 and shRNA-4, or a control shRNA; the RIPK4 protein level as measured by western blot. **b** Wound healing assays showing that *RIPK4*-silenced T24 and RT4 cells have lower motility compared to that of the control cells. **c** Matrigel invasion assays showing that *RIPK4*-silenced T24 and RT4 cells have decreased invasive capacity compared to that of the control cells. **d** Representative images of human umbilical vein endothelial cells (HUVECs) cultured with conditioned medium derived from *RIPK*4-silenced cells and control shNC cells. **e** Silencing of *RIPK4* suppressed T24 and RT4 cell invasion and metastasis in severe combined immunodeficient (SCID-Beige) mice in vivo. Upper panel: Examples of haematoxylin and eosin staining in four lung nodule samples originating from T24-vector, T24-shRIPK4, RT4-vector, RT4-shRIPK4 cell-injected mice. Original magnification, ×200. Lower panel: Number of metastases in the lungs of mice (*n* = 7 per group) 8 weeks after tail injection of scrambled control shRNA T24 and RT4 cells (red; mean ± SEM, 13.3 ± 4.1 for T24, 14.4 ± 7.0 for RT4), and *RIPK4* shRNA T24 and RT4 cells (blue; mean ± SEM, 6.2 ± 2.8 for T24, 3.4 ± 2.8 for RT4). The nodules were examined under an anatomical microscope. **f** Ectopic expression of *RIPK4* was substantially increased in BIU87-RIPK4 cells compared with that in BIU87-vector cells by western blot. **g** Wound healing assays demonstrating that BIU87-RIPK4 cells had higher motility than BIU87-vector cells. **h** Ectopic overexpression of *RIPK4* enhanced BIU87 cell invasion in a transwell assay. **I** Representative images of human umbilical vein endothelial cells (HUVECs) cultured with conditioned medium derived from *RIPK4*-overexpressing cells and vector control cells. **j** Overexpression of *RIPK4* promoted BIU87 cell invasion and metastasis in severe combined immunodeficient (SCID-Beige) mice in vivo. Upper panel: Examples of haematoxylin and eosin staining in four lung nodule samples originating from BIU87-vector and BIU87-RIPK4 cell-injected mice. Original magnification, ×200. Lower panel: Number of metastases in lungs of mice (*n* = 7 per group) 8 weeks after tail injection of BIU87-vector cells (red; mean ± SEM, 3.0 ± 2.2) and BIU87-RIPK4 cells (blue; mean ± SEM, 11.1 ± 4.9). The nodules were examined under an anatomical microscope. *P < 0.05, **P < 0.01 by Student’s *t* test

To determine the function of RIPK4 in BC invasion and metastasis, we constructed several stable cell lines transfected with shRNAs or the negative control shNC into T24 and RT4 cells. Among the four RIPK4 shRNAs tested, ShRNA-3 generated the most consistent knockdown results in T24 and RT4 cell lines and was thus chosen for further studies (Fig. 2a). The wound healing and matrigel invasion assays indicated that *RIPK4* knockdown by ShRNA-3 transfection reduced the migration and invasion abilities of T24 and RT4 cells significantly compared with the control shNC cells (Fig. 2b, c). In addition, conditioned medium from *RIPK4*-shRNA T24 and RT4 cells exhibited a decreased ability to induce tubule formation by HUVECs (Fig. 2d). More importantly, using a lung metastasis in vivo animal model, the sizes and numbers of metastatic nodules were significantly decreased after *RIPK4* knockdown in both T24 and RT4 cells (Fig. 2e). These results implied that knockdown of *RIPK4* suppresses the invasiveness and metastasis of BC cells.

### RIPK4 overexpression promotes BC cell invasion and metastasis in vitro and in vivo

To evaluate the function of RIPK4 in BC progression further, we transiently expressed *RIPK4* in BC cell lines BIU87. By western blotting, high levels of the RIPK4 protein were found in the BIU87-RIPK4 stable cells, whereas it was present at a low level in the BIU87-Vector control stable cell line (Fig. 2f). BIU87 cells’ migration and invasion capabilities were increased dramatically by overexpression of *RIPK4* (Fig. 2g, h). Additionally, conditioned medium from *RIPK4*-transduced BIU87 cells showed an increased capacity to induce tubule formation by HUVECs (Fig. 2i). Furthermore, using the lung metastasis in vivo animal model, the sizes and numbers of metastatic nodules increased dramatically after *RIPK4* overexpressed in BIU87 cells (Fig. 2j). These results were consistent with the *RIPK4* knockdown data and showed that RIPK4 promotes invasiveness and metastasis of BC cells.

### Correlation between EMT markers and the expression of RIPK4 in BC

To investigate the relationship between RIPK4 and EMT, after silencing *RIPK4* in T24 and RT4 cells, the protein levels of two epithelial markers, E-cadherin and β-catenin, were increased, whereas the levels of two mesenchymal markers, fibronectin and vimentin, were decreased, as shown by both western blotting (Supplementary Figure S1A and C) and immunofluorescence staining (Supplementary Figure S1B and D). By contrast, following ectopic overexpression of *RIPK4* in BIU87 cells, the E-cadherin and β-catenin levels decreased, while those of fibronectin and vimentin increased, as shown by western blotting (Supplementary Figure S1E) and immunofluorescence staining (Supplementary Figure S1F). Furthermore, IHC staining was performed for 112 primary BC tissues. Representative IHC staining of EMT markers in BC tissues is displayed in Supplementary Figure S1G. The RIPK4 level correlated positively with the level of mesenchymal markers: vimentin (*P* < 0.001), fibronectin (*P* = 0.002), and correlated negatively with the epithelial markers: E-cadherin (*P* < 0.001), β-catenin (*P* < 0.001) (Supplementary Table S4). These data illustrated that in BC progression, RIPK4 contributed to the EMT process.

### RIPK4 upregulated VEGF-A expression in BC

**Fig. 3:**
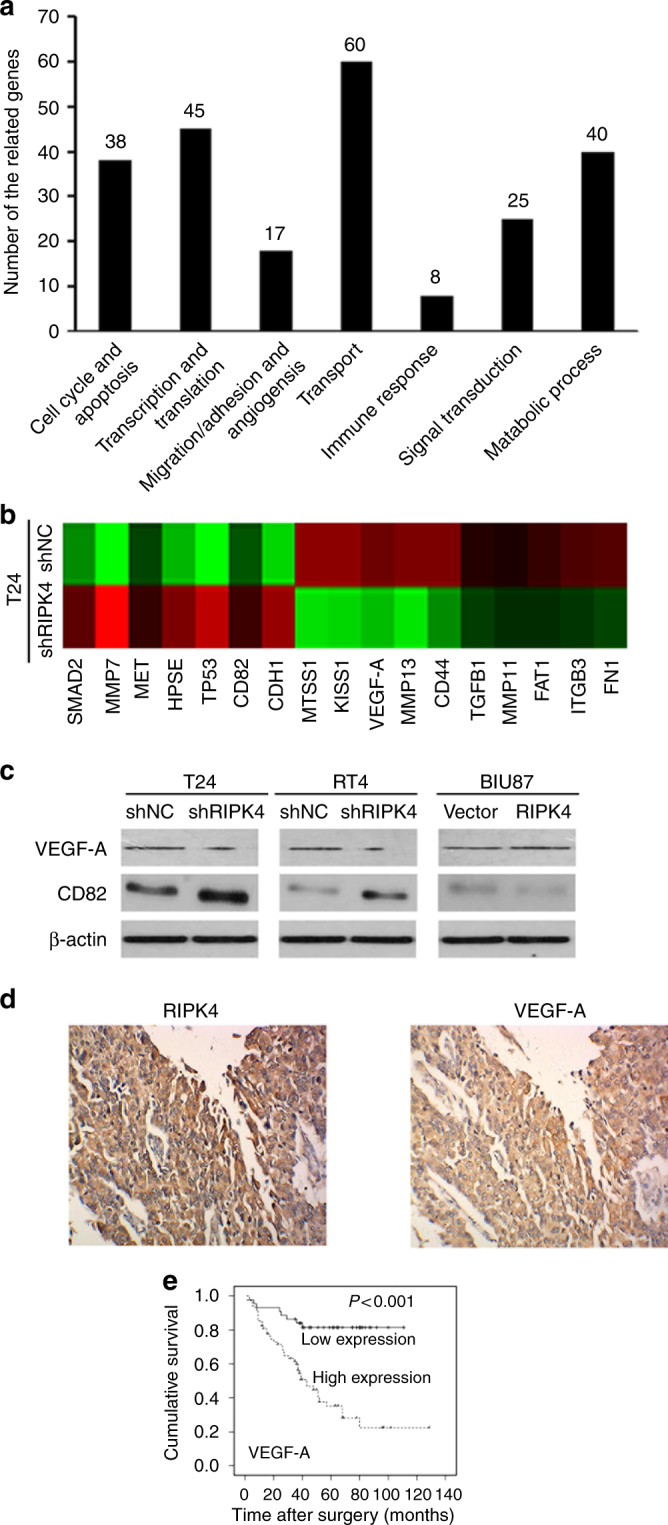
RIPK4 regulates VEGF-A expression in BC cells and tissues. **a** Using DNA microarray experiments, the differentially expressed genes (>twofold, *P* < 0.001) resulting from transfection with T24-shRIPK4 compared with transfection with the control T24-shNC were categorised according to their functions. The number of genes in each category is bracketed. **b** Seventeen genes, *CDH1*, *CD82*, *HPSE*, *MMP7*, *MET*, *TP53*, *SMAD2*, *CD44*, *FN1*, *FAT1*, *ITGB3*, *KISS1*, *MMP11*, *MMP13*, *VEGF-A*, *TGFB1*, and *MTSS1*, showed more than a twofold mRNA differential expression in T24-shRIPK4 cells compared with that in T24-shNC cells related to migration/angiogenesis. **c** Western blot analysis showed that *RIPK4* silenced by shRNA transfection leads to reduced levels of VEGF-A and increased levels of CD82 in T24 and RT4 cells; whereas ectopic overexpression of *RIPK4* by pcDNA-RIPK4 transfection had the reverse effects on VEGF-A and CD82 in BIU87 cells. **d** Representative immunohistochemistry images showing the high expression of RIPK4 and high expression of VEGF-A in a representative BC tissue sample. Original magnification, ×200. The levels of RIPK4 and VEGF-A are correlated positively (*P* = 0.005). **e** Upregulation of VEGF-A is significantly associated with poorer survival in patients with BC

To further investigate the underlying molecular mechanisms of RIPK4-mediated cell migration/invasion in BC, a DNA microarray was used to compare the global gene expression profiles of T24 cells transfected with shRNA RIPK4 and shNC. Among 233 differentially expressed genes (over twofold change; *P* < 0.001), 97 were upregulated and 136 were downregulated (Fig. 3a). Among them were 17 genes (*CDH1*, *CD82*, *HPSE*, *MMP7*, *MET*, *TP53*, *SMAD2*, *CD44*, *FN1*, *FAT1*, *ITGB3*, *KISS1*, *MMP11*, *MMP13, VEGF-A, TGFB1* and *MTSS1*) related to migration and/or angiogenesis (Fig. 3b and Supplementary Table S5). Western blotting also showed that knocking down *RIPK4* using shRIPK4 transfection downregulated the VEGF-A level and increased the abundance of CD82 in T24 and RT4 cells significantly (Fig. 3c). Consistently, overexpression of *RIPK4* by pcDNA-RIPK4 transfection significantly upregulated the VEGF-A level and decreased the CD82 level in BIU87 cells (Fig. 3c). Additionally, we performed IHC staining for RIPK4 and VEGF-A on the 112 BC specimens. The results showed that the RIPK4 level correlated positively with the VEGF-A level in this cohort of BC tissues (*P* = 0.005, Fig. 3d and Supplementary Table S6). However, there were no significant differences in CD82 levels between the RIPK4 low and high groups (*P* = 0.411, Supplementary Table S6). Importantly, we observed an association between higher VEGF-A levels and poor survival in the BC cohort (*P* < 0.001, Fig. 3e), which was consistent with that of the higher RIPK4 levels in BC specimens. These results suggested that VEGF-A has a major role in RIPK4-promoted invasion and metastasis in BC.

### VEGF-A mediates RIPK4-induced BC cell EMT and invasion/metastasis

**Fig. 4:**
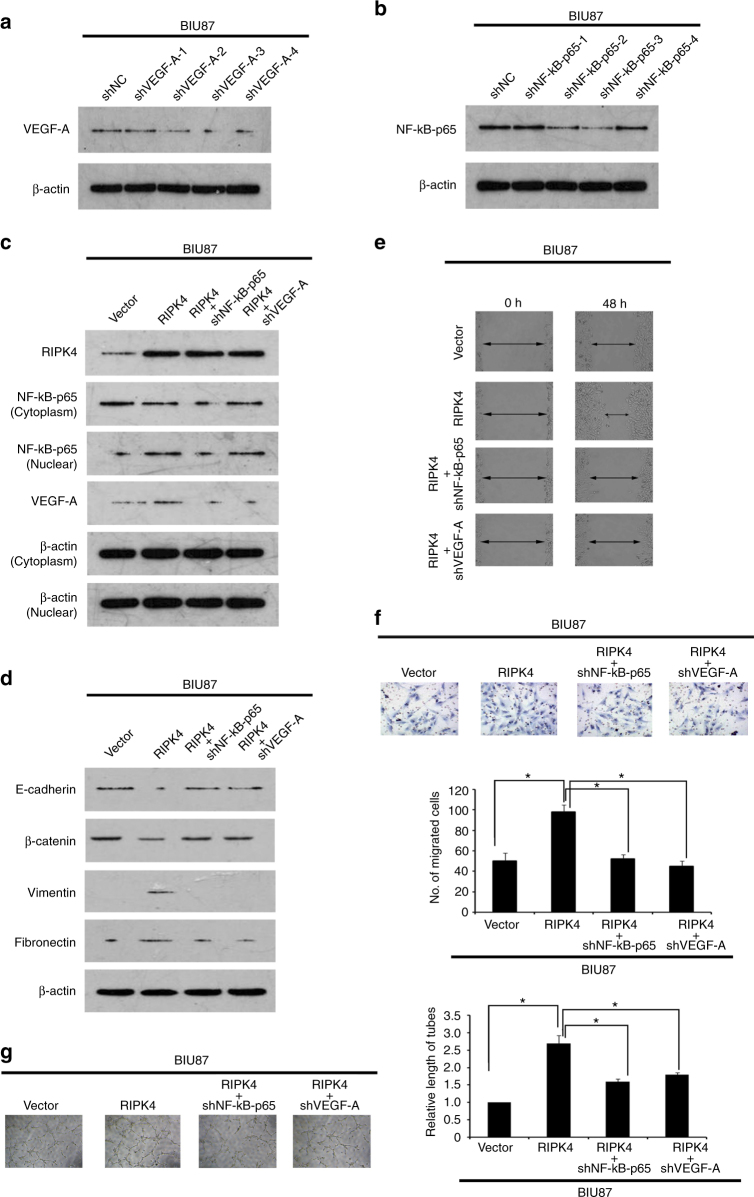
RIPK4-mediated BC BIU87 cell EMT and invasion/migration are partly inhibited after silencing of *VEGF-A* or *NF-κB-p65* by specific shRNAs. **a** BIU87 cells were infected with lentivirus-expressing *VEGF-A* shRNA-1, shRNA-2, shRNA-3 and shRNA-4, or a control shRNA, VEGF-A protein levels were measured by western blot. **b** BIU87 cells were infected with lentivirus-expressing *NF-κB-p65* shRNA-1, shRNA-2, shRNA-3 and shRNA-4, or a control shRNA, the NF-κB-p65 protein level was measured by western blot. **c** Western blot showing that after silencing of *VEGF-A* or *NF-κB-p65* in RIPK4-BIU87 cells, the levels of the nuclear NF-κB-p65 and VEGF-A decreased. β-actin was used as a loading control. **d** Western blot showing that after silencing of *VEGF-A* or *NF-κB-p65* in RIPK4-BIU87 cells, the levels of the mesenchymal markers (vimentin and fibronectin) decreased and the levels of the epithelial markers (E-cadherin and β-catenin) increased. β-actin was used as a loading control. **e** Wound healing assays showing that the enhanced migratory ability of RIPK4-BIU87 cells was inhibited by silencing of *VEGF-A* or *NF-κB-p65*. **f** The invasive ability of RIPK4-BIU87 cells was inhibited dramatically after shVEGF-A or shNF-κB-p65 treatment in a transwell assay. Data are the means ± SE of three independent experiments. **g** Representative images of human umbilical vein endothelial cells (HUVECs) cultured with conditioned medium derived from the indicated cells. *P < 0.05 by Student’s *t* test

To investigate if VEGF-A is required for RIPK4-induced EMT and invasion/metastasis in BC cell, *VEGF-A* expression in BIU87-RIPK4 cells was silenced using shRNAs (Fig. 4a, c). ShVEGF-A treatment inhibited RIPK4-induced EMT, as shown by the increased abundance of epithelial markers (E-cadherin and β-catenin) and the decreased levels of mesenchymal markers (vimentin and fibronectin) in BIU87-RIPK4 cells (Fig. 4d and Supplementary Figure S2). In addition, wound healing (Fig. 4e), transwell assays (Fig. 4f) and HUVECs tubule formation assays (Fig. 4g) indicated that the migratory and invasive abilities of BIU87-RIPK4 cells were inhibited dramatically after *VEGF-A* was silenced. Taken together, these data provided evidence that VEGF-A mediates the RIPK4-induced EMT and invasion/metastasis in BC cells.

### NF-κB signalling is activated by RIPK4 overexpression

**Fig. 5:**
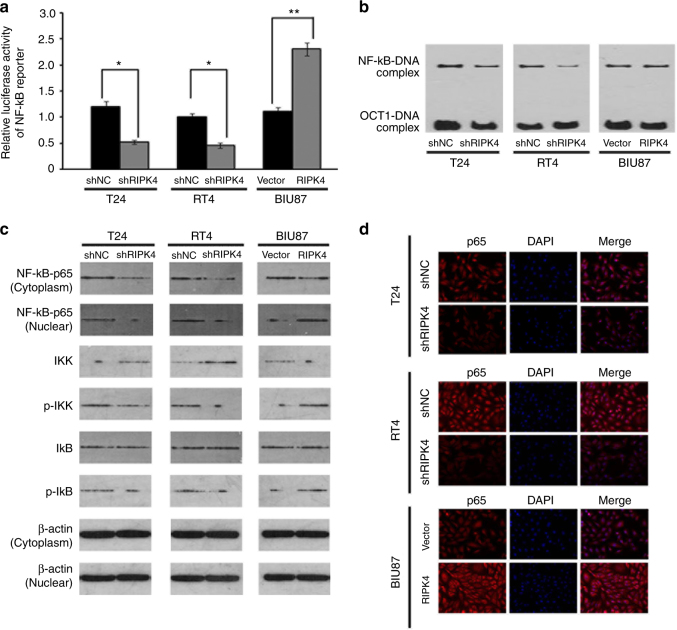
Overexpressing *RIPK4* activates NF-κB signalling. **a** NF-κB luciferase reporter activities were analysed in the indicated cells. Each bar represents the mean ± SD of three independent experiments. **b** EMSA indicating that endogenous NF-κB activity increased in *RIPK4*-transduced cells and decreased in *RIPK4*-silenced cells. The OCT-1-DNA binding complex served as a control. **c** Western blot of nuclear NF-κB-p65, cytoplasmic NF-κB-p65, IKK, p-IKK, IκB and p-IκB levels in the indicated cells. β-actin was used as a loading control. **d** Immunofluorescence staining showing reduced levels of p65 in shRIPK4 T24 and RT4 cells relative to the control shNC T24 and RT4 cells, and increased levels of p65 in BIU87-RIPK4 cells compared with BIU87-vector cells. *P < 0.05, **P < 0.01 by Student’s *t* test

NF-κB is a key regulator of the transcription of *VEGF-A*;^26^ therefore, we investigated the involvement of RIPK4 in the regulation of the NF-κB pathway in BC. Overexpression of *RIPK4* increased the activity of the NF-κB luciferase reporter gene significantly, whereas silencing of *RIPK4* reduced the reporter activity significantly (Fig. 5a). Importantly, the nuclear levels NF-κB-p65 were decreased dramatically in *RIPK4*-silenced cells but increased in *RIPK4*-transduced cells (Fig. 5b, c). By contrast, after the ectopic overexpression of *RIPK4* in BIU87 cells, the level of NF-κB-p65 increased, whereas silencing of *RIPK4* decreased the amount of NF-κB-p65, as evidenced by immunofluorescence staining (Fig. 5d).

To further validate that RIPK4-mediated BC EMT and that invasion/metastasis occur via NF-κB activation, the NF-κB pathway was blocked in RIPK4-overexpressing cells using an shRNA targeting the gene encoding NF-κB-p65 (*RELA*), shRNA-NF-κB-p65 (Fig. 4b, c). As expected, *RIPK4* overexpression’s stimulatory effect on NF-κB activation was inhibited by shRNA-NF-κB-p65 (Fig. 4c). Additionally, RIPK4-induced EMT was also inhibited by shRNA-NF-κB-p65, as shown by increased levels of epithelial markers (E-cadherin and β-catenin) and decreased levels of mesenchymal markers (vimentin and fibronectin) in BIU87-RIPK4 cells (Fig. 4d and Supplementary Figure S2). Furthermore, shRNA-NF-κB-p65 decreased RIPK4-induced invasion and metastasis, as evidenced by wound healing (Fig. 4e), transwell assays (Fig. 4f) and HUVECs tubule formation assays (Fig. 4g). Taken together, our results suggested that RIPK4’s oncogenic activity is dependent on activation of NF-κB.

### RIPK4 maintains NF-κB activation

**Fig. 6:**
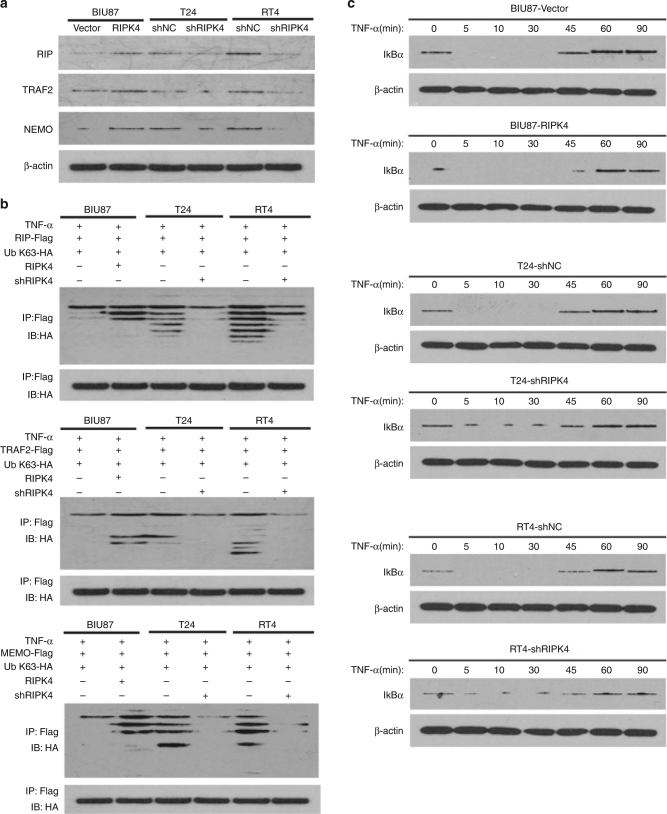
RIPK4 sustains NF-κB activation. **a** RIPK4 stabilises TRAF2, RIP and NEMO in BC cells. **b** Western blot analysis of the K63-linked polyubiquitination levels of RIP (upper panel), TRAF2 (middle panel) and NEMO (lower panel) in the indicated cells treated with TNF-α (10 ng/mL). **c** Western blot analysis of IκBα expression in the indicated cells treated with TNF-α (10 ng/mL); β-actin was used as a loading control

Stimulation of tumour necrosis factor (TNF) induces the rapid recruitment and ubiquitination of RIP, TRAF2 and NEMO in the NF-κB receptor complex, which process is crucial for TNF-induced NF-κB activation.^27,28^ We observed significantly increased levels of RIP, TRAF2 and NEMO in cell membranes isolated from *RIPK4*-overexpressing cells, compared with vector control cells, and the levels of the same proteins isolated from the cell membranes of *RIPK4*-silenced cells were decreased significantly compared to RNAi vector-transformed cells (Fig. 6a). Figure 6b shows that the K63-polyubiquitination levels of RIP, TRAF2,and NEMO were higher in *RIPK4*-transduced cells and lower in *RIPK4*-silenced cells compared with the corresponding control cells. This suggested that in NF-κB signalling, RIPK4 promotes ubiquitin conjugation. The NF-κB signalling pathway can be subdivided according to the different inducers, such as TNF and IL-1β; therefore, we further observed that the decreased levels of IκB induced by TNF-α treatment were prolonged significantly in *RIPK4*-overexpressing cells and reduced in *RIPK4*-silenced cells (Fig. 6c). These results suggested that RIPK4 sustains NF-κB activation by promoting ubiquitin conjugation of NF-κB signalling-related proteins.

### Clinical relevance of RIPK4-induced NF-kB activation in human BC

Finally, we investigated the clinical relevance of RIPK4’s activation NF-κB in BC cells. The EMSA and qRT-PCR result showed that RIPK4 levels correlated positively with NF-κB activity, and with the mRNA levels of the NF-κB downstream gene *VEGF-A* in human BCs (Supplementary Figure S3A). Consistently, IHC analysis of 112 BC tissue specimens showed that RIPK4 levels correlated positively with the p-p65 levels (*P* < 0.001; Supplementary Figure S3B and Supplementary Table S6). Importantly, positivity for p-p65 was associated with poor survival in this BC cohort (*P* = 0.001, Supplementary Figure S3C). Furthermore, based on the results derived from GEPIA (http://gepia.cancerpku.cn), which could analyse the RNA-sequencing expression data of 23 types of cancers and normal samples from the TCGA according to the standard processing pipeline,^29,30^ RIPK4 levels were significantly correlated with VEGF-A expression in BC (*r* = 0.2, *P* = 5.5e−05), melanoma (*r* = 0.45, *P* = 3.5e−05) and thymoma (*r* = 0.73, *P* = 9.7e−21) (Supplementary Figure S4). These data supported our hypothesis that *RIPK4* overexpression activates the NF-κB signalling pathway, ultimately leading to an aggressive BC phenotype and poor clinical outcomes for patients with BC.

## Discussion

NF-κB is an important transcription factor that is activated in BC and has a vital role in tumour progression.^31,32^ During the progression of BC, NF-κB signalling modulates several key biological processes by inducing the transcription of several genes that regulate cell proliferation, survival, invasion and angiogenesis.^33^ In addition, downstream targets of NF-κB, such as VEGF, and the nuclear expression and activity of NF-κB are increased significantly in BC tissues compared with normal bladder urothelial tissues.^8,34,35^ BCs with highly active NF-κB also show aggressive pathological features and have poor treatment outcomes.^36^ Importantly, blocking the NF-κB pathway inhibited BC metastasis and angiogenesis.^37,38^ These studies indicated that activation of NF-κB is important in BC progression. Accordingly, improving our understanding of NF-κB signalling regulation might identify novel targets for BC therapy. In the present study, RIPK4 was observed to promote NF-κB activity by the recruitment and ubiquitination of RIP. Silencing *RIPK4* dramatically reduced, whereas overexpressing *RIPK4* promoted, NF-κB activity. Consequently, the results revealed a novel mechanism of NF-κB activation in BCs.

RIPK4, containing an N-terminal RIP-like kinase domain and a C-terminal region characterised by 11 ankyrin repeats, is a member of a Ser/Thr kinase family that regulates signal transduction.^19^ Until now, RIPK4’s function in the pathogenesis of malignant diseases has not been investigated extensively. Liu and colleagues reported that RIPK4 is overexpressed in cervical squamous cell cancer, and *RIPK4* knockdown in vitro reduced cell migration and invasion.^17^ Similarly, RIPK4 is overexpressed in skin, ovarian and colorectal cancers.^16^ However, Adams et al. found that RIPK4 is downregulated in keratinocytes of the hyperproliferative epithelium;^39^ in hepatocellular carcinoma and tongue squamous cell cancer, RIPK4 expression is also reported to be downregulated.^40,41^ The discordance among these studies suggested that in different tumours, RIPK4 has different carcinogenic mechanisms. To explore the utility of RIPK4 as a therapeutic target in BC, we observed that RIPK4 levels were increased in BC cell lines and in primary BC tissues, and were correlated positively with progression and patient survival in BC. Overexpressing *RIPK4* promoted, but silencing RIPK4 reduced, BC EMT and invasion/metastasis. Thus, our results suggested that RIPK4 functions as an oncogene in BCs, and RIPK4 overexpression contributes to BC progression.

However, the molecular mechanisms underlying RIPK4’s regulation of BC cell migration/invasion are unclear. To obtain a deeper understanding of the downstream molecular processes involving RIPK4 and the invasiveness and metastasis of BC, the global gene expression profiles between T24 cells transfected with shRIPK4 or shNC were compared using DNA microarray technology. Among the differentially expressed genes associated with migration and angiogenesis, 17 were differentially expressed by ≫twofold. Subsequently, the protein levels of these 17 genes were analysed using western blotting. This downregulation of VEGF-A was validated at the protein level using western blotting in *RIPK4*-shRNA T24 and RT4 cells. By contrast, overexpression of *RIPK4* increased the protein levels of VEGF-A in BIU87 cells. In addition, we observed a significant and positive correlation between the RIPK4 levels and VEGF-A levels in our cohort of BC tissues. Collectively, our results implied that RIPK4 might regulate cell migration and invasion via VEGF-A in BC cells.

Recently, angiogenesis was identified an effective and important target of pharmacological strategies against BC; for example, using monoclonal antibodies against VEGF and the VEGF receptor.^42^ There is an urgent need to discover new and improved anti-angiogenic agents to treat for patients with BC. In the present study, we showed that overexpression of *RIPK4* resulted in the upregulation of certain known pro-angiogenic factors, such as VEGF-A, in BC cells. This indicated that RIPK4 could represent an attractive target for BC therapy. Therefore, the correlation between RIPK4 levels in the blood of patients with BC and their survival should be evaluated further.

Accumulating evidence demonstrates that the expression of several VEGF genes (including *VEGF-A*) is by upregulation of the p65 subunit and by nuclear translocation-induced NF-κB activation in many human cancers.^43–45^ In addition, Meylan and colleagues showed dose-dependent activation of NF-κB in 293T cells by the overexpression of *RIPK4*.^19^ Kim et al. also demonstrated that *RIPK4* upregulation in various diffuse large B-cell lymphoma cell lines caused activation of NF-κB, whereas *RIPK4* inactivation led to a suppression of NF-κB, increased survival in vitro and sensitisation of these cell lines cells to treatment with chemotherapeutic agents.^18^ Our results were consistent with previous studies, suggesting that RIPK4 has an important function in the activation of NF-κB. Moreover, we hypothesised that NF-κB activation in response to RIPK4 is stimulated mainly by the recruitment and ubiquitination of RIP. Our results showed that RIPK4 has a large impact on NF-κB pathway activation, suggesting that an NF-κB-regulated gene may have differing molecular mechanisms in different cell types, which is likely to depend on the specific context of the cells. The mechanism underlying RIPK4’s activation and maintenance of NF-κB activity is under investigation in our laboratory.

In summary, we reported the RIPK4 expression pattern in BC and demonstrated the potential role of RIPK4 in cancer aggression. In addition, functional and mechanistic studies of RIPK4 suggested its critical function in cell EMT, invasion and metastasis by upregulating VEGF-A through the NF-κB pathway. We also found that the level RIPK4 correlated with level of VEGF-A in BC. Combined with the data from previous studies, the results presented here provided strong evidence that RIPK4 dysregulation has important roles in many pathological processes. Moreover, our results showed that high expression of RIPK4 is a novel and independent prognostic factor for patients with BC, enabling clinicians to identify high-risk patients that require more intensive treatment. Thus, targeting the RIPK4 pathway might represent a new therapeutic strategy to improve the therapy and survival of patients with BC or other cancers. Further study of the mechanisms of RIPK4 expression in human cancers is also required to improve our understanding of the biological basis of cancer progression.

## Electronic supplementary material

Supplementary figure legends

Supplementary Figure S1

Supplementary Figure S2

Supplementary Figure S3

Supplementary Figure S4

Supplementary Table S1

Supplementary Table S2

Supplementary Table S3

Supplementary Table S4

Supplementary Table S5

Supplementary Table S6

Supplementary materials and methods

## References

[1] Chen W (2016). Cancer statistics in China, 2015. CA Cancer J. Clin..

[2] Gui Y (2011). Frequent mutations of chromatin remodeling genes in transitional cell carcinoma of the bladder. Nat. Genet..

[3] Kaplan AL, Litwin MS, Chamie K (2014). The future of bladder cancer care in the USA. Nat. Rev. Urol..

[4] Vashistha V, Quinn DI, Dorff TB, Daneshmand S (2014). Current and recent clinical trials for perioperative systemic therapy for muscle invasive bladder cancer: a systematic review. BMC Cancer.

[5] Alfred Witjes J (2017). Updated 2016 EAU guidelines on muscle-invasive and metastatic bladder cancer. Eur. Urol..

[6] Fu YP (2014). The 19q12 bladder cancer GWAS signal: association with cyclin E function and aggressive disease. Cancer Res..

[7] Huang Z (2015). Down-regulation of HMGB1 expression by shRNA constructs inhibits the bioactivity of urothelial carcinoma cell lines via the NF-kappaB pathway. Sci. Rep..

[8] Lee SJ, Lim JH, Choi YH, Kim WJ, Moon SK (2012). Interleukin-28A triggers wound healing migration of bladder cancer cells via NF-kappaB-mediated MMP-9 expression inducing the MAPK pathway. Cell Signal..

[9] Mukherjee N, Houston TJ, Cardenas E, Ghosh R (2015). To be an ally or an adversary in bladder cancer: the NF-kappaB story has not unfolded. Carcinogenesis.

[10] Hattori M (2000). The DNA sequence of human chromosome 21. Nature.

[11] Holland P (2002). RIP4 is an ankyrin repeat-containing kinase essential for keratinocyte differentiation. Curr. Biol..

[12] Adams S, Munz B (2010). RIP4 is a target of multiple signal transduction pathways in keratinocytes: implications for epidermal differentiation and cutaneous wound repair. Exp. Cell Res..

[13] Rountree RB (2010). RIP4 regulates epidermal differentiation and cutaneous inflammation. J. Invest. Dermatol..

[14] Kalay E (2012). Mutations in RIPK4 cause the autosomal-recessive form of popliteal pterygium syndrome. Am. J. Hum. Genet..

[15] Mitchell K (2012). Exome sequence identifies RIPK4 as the Bartsocas-Papas syndrome locus. Am. J. Hum. Genet..

[16] Huang X (2013). Phosphorylation of dishevelled by protein kinase RIPK4 regulates Wnt signaling. Science.

[17] Liu DQ (2015). Increased RIPK4 expression is associated with progression and poor prognosis in cervical squamous cell carcinoma patients. Sci. Rep..

[18] Kim SW (2008). Protein kinase C-associated kinase is required for NF-kappaB signaling and survival in diffuse large B-cell lymphoma cells. Blood.

[19] Meylan E, Martinon F, Thome M, Gschwendt M, Tschopp J (2002). RIP4 (DIK/PKK), a novel member of the RIP kinase family, activates NF-kappa B and is processed during apoptosis. EMBO Rep..

[20] Liu JY (2013). PinX1 suppresses bladder urothelial carcinoma cell proliferation via the inhibition of telomerase activity and p16/cyclin D1 pathway. Mol. Cancer.

[21] Suzuki M (2016). Targeting ceramide synthase 6-dependent metastasis-prone phenotype in lung cancer cells. J. Clin. Invest..

[22] Yan LX (2008). MicroRNA miR-21 overexpression in human breast cancer is associated with advanced clinical stage, lymph node metastasis and patient poor prognosis. RNA.

[23] Smith BD, Smith GL, Carter D, Sasaki CT, Haffty BG (2000). Prognostic significance of vascular endothelial growth factor protein levels in oral and oropharyngeal squamous cell carcinoma. J. Clin. Oncol..

[24] Buchholz TA (2005). The nuclear transcription factor kappaB/bcl-2 pathway correlates with pathologic complete response to doxorubicin-based neoadjuvant chemotherapy in human breast cancer. Clin. Cancer Res..

[25] Christgen M (2008). KAI1/CD82 is a novel target of estrogen receptor-mediated gene repression and downregulated in primary human breast cancer. Int. J. Cancer.

[26] Karin M, Greten FR (2005). NF-kappaB: linking inflammation and immunity to cancer development and progression. Nat. Rev. Immunol..

[27] Ea CK, Deng L, Xia ZP, Pineda G, Chen ZJ (2006). Activation of IKK by TNFalpha requires site-specific ubiquitination of RIP1 and polyubiquitin binding by NEMO. Mol. Cell.

[28] Wang C (2001). TAK1 is a ubiquitin-dependent kinase of MKK and IKK. Nature.

[29] Tang Z (2017). GEPIA: a web server for cancer and normal gene expression profiling and interactive analyses. Nucleic Acids Res..

[30] Chen WJ (2017). Clinical roles of the aberrantly expressed lncRNAs in lung squamous cell carcinoma: a study based on RNA-sequencing and microarray data mining. Oncotarget.

[31] Hayden MS, Ghosh S (2004). Signaling to NF-kappaB. Genes Dev..

[32] Kang S (2005). Polymorphism in the nuclear factor kappa-B binding promoter region of cyclooxygenase-2 is associated with an increased risk of bladder cancer. Cancer Lett..

[33] Karashima T (2003). Nuclear factor-kappaB mediates angiogenesis and metastasis of human bladder cancer through the regulation of interleukin-8. Clin. Cancer Res..

[34] Rayet B, Gelinas C (1999). Aberrant rel/nfkb genes and activity in human cancer. Oncogene.

[35] Degoricija M (2014). High NF-kappaB and STAT3 activity in human urothelial carcinoma: a pilot study. World J. Urol..

[36] Levidou G (2008). Clinical significance of nuclear factor (NF)-kappaB levels in urothelial carcinoma of the urinary bladder. Virchows. Arch..

[37] Karin M, Cao Y, Greten FR, Li ZW (2002). NF-kappaB in cancer: from innocent bystander to major culprit. Nat. Rev. Cancer.

[38] Wang CY, Cusack JC, Liu R, Baldwin AS (1999). Control of inducible chemoresistance: enhanced anti-tumour therapy through increased apoptosis by inhibition of NF-kappaB. Nat. Med..

[39] Adams S, Pankow S, Werner S, Munz B (2007). Regulation of NF-kappaB activity and keratinocyte differentiation by the RIP4 protein: implications for cutaneous wound repair. J. Invest. Dermatol..

[40] Heim D (2015). Retroviral insertional mutagenesis in telomerase-immortalised hepatocytes identifies RIPK4 as novel tumor suppressor in human hepatocarcinogenesis. Oncogene.

[41] Wang X, Zhu W, Zhou Y, Xu W, Wang H (2014). RIPK4 is downregulated in poorly differentiated tongue cancer and is associated with migration/invasion and cisplatin-induced apoptosis. Int. J. Biol. Markers.

[42] Vogelzang NJ (2013). Antiangiogenic agents, chemotherapy, and the treatment of metastatic transitional cell carcinoma. J. Clin. Oncol..

[43] Kinose Y (2015). IKKbeta regulates VEGF expression and is a potential therapeutic target for ovarian cancer as an antiangiogenic treatment. Mol. Cancer Ther..

[44] Noort AR (2014). NF-kappaB-inducing kinase is a key regulator of inflammation-induced and tumour-associated angiogenesis. J. Pathol..

[45] Yokoi K (2005). Simultaneous inhibition of EGFR, VEGFR, and platelet-derived growth factor receptor signaling combined with gemcitabine produces therapy of human pancreatic carcinoma and prolongs survival in an orthotopic nude mouse model. Cancer Res..

