# Supplementary material for: RIPK4 promotes bladder urothelial carcinoma cell aggressiveness by upregulating VEGF-A through the NF-κB pathway
Source: Br J Cancer. 2018 Jun 5;118(12):1617–27. doi: 10.1038/s41416-018-0116-8 (PMC6008479; doi:10.1038/s41416-018-0116-8)
Supplement: Supplementary file 1 — Supplementary figure legends [file 41416_2018_116_MOESM1_ESM.docx]

**Supplementary figure legends**

**Supplementary Figure S1. Correlation between the expression of RIPK4 and EMT markers in BC cell lines and tissues. (A, B)** Western blot and immunofluorescence staining assays revealing that knockdown of *RIPK4* by shRIPK4 resulted in increased levels of epithelial makers (E-cadherin and β-catenin) and decreased levels of mesenchymal markers (vimentin and fibronectin) in T24 cells, compared with those in control shRNA-treated cells. **(C, D)** Western blot and immunofluorescence staining assays revealing that knockdown of *RIPK4* by shRIPK4 resulted in increased levels of epithelial makers (E-cadherin and β-catenin) and decreased levels of mesenchymal markers (vimentin and fibronectin) in RT4 cells, compared with that in control shRNA-treated cells. **(E, F)** Western blot and immunofluorescence staining assays showing decreased levels of the epithelial markers (E-cadherin and β-catenin) and increased levels of the mesenchymal markers (vimentin and fibronectin) in BIU87-RIPK4 cells compared with that in BIU87-vector cells. **(G)** Immunohistochemical staining showing that high levels of RIPK4 in BC tissues was accompanied by decreased levels of E-cadherin and β-catenin and increased levels of vimentin and fibronectin. Original magnification, ×200.

**Supplementary Figure S2. *VEGF-A* and *NF-κB-p65* is responsible for RIPK4-induced BC cell EMT. (A)** Immunofluorescence staining showing that after silencing of *VEGF-A* or *NF-κB-p65* in RIPK4-BIU87 T24 cells, the levels of E-cadherin increased. **(B)** Immunofluorescence staining showing that after silencing of *VEGF-A* or *NF-κB-p65* in RIPK4-BIU87 T24 cells, the levels of β-catenin increased. **(C)** Immunofluorescence staining showing that after silencing of *VEGF-A* or *NF-κB-p65* in RIPK4-BIU87 T24 cells, the levels of vimentin decreased. **(D)** Immunofluorescence staining showing that after silencing of *VEGF-A* or *NF-κB-p65* in RIPK4-BIU87 T24 cells, the levels of fibronectin decreased.

**Supplementary Figure S3.** **Clinical relevance of the RIPK4/NF-κB/VEGF-A axis in human BCs. (A)** EMSA of the NF-κB-DNA complex; western blot of RIPK4 expression; and qRT-PCR analysis of VEGF-A mRNA expression in clinical samples. **(B)** RIPK4 levels were associated positively with NF-κB-p65 expression in 112 primary human BC specimens. Two representative cases are shown. Original magnification, ×200. **(C)** Positive expression of p-p65 is significantly associated with poorer survival in patients with BC.

**Supplementary Figure S4. Analysis between RIPK4 and VEGF-A mRNA expression from GEPIA.** **(A)** RIPK4 levels were associated positively with VEGF-A expression in BC. **(B)** RIPK4 levels were associated positively with VEGF-A expression in melanoma. **(C)** RIPK4 levels were associated positively with VEGF-A expression in thymoma.
