# Supplementary material for: RIPK4 promotes bladder urothelial carcinoma cell aggressiveness by upregulating VEGF-A through the NF-κB pathway
Source: Br J Cancer. 2018 Jun 5;118(12):1617–27. doi: 10.1038/s41416-018-0116-8 (PMC6008479; doi:10.1038/s41416-018-0116-8)
Supplement: Supplementary file 6 — Supplementary Table S1 [file 41416_2018_116_MOESM6_ESM.doc]

| **Supplementary Table S1.** Correlation between RIPK4 and the clinicopathological features of BC | | | | |
| --- | --- | --- | --- | --- |
|  |  | Expression of RIPK4 | |  |
| Characteristics | No of patients (n = 112) | Low(%) (n = 58) | High(%) (n = 54) | *P* value^a^ |
| Age (years) |  |  |  | 0.725 |
| ≤67^b^ | 60 | 32(53.3) | 28(46.7) |  |
| ＞67 | 52 | 26(50.0) | 26(50.0) |  |
| Gender |  |  |  | 0.852 |
| Male | 90 | 47(52.2) | 43(47.8) |  |
| Female | 22 | 11(50.0) | 11(50.0) |  |
| Tumor size (cm) |  |  |  | 0.134 |
| ≤3.6^c^ | 58 | 34(58.6) | 24(41.4) |  |
| ＞3.6 | 54 | 24(44.4) | 30(55.6) |  |
| Tumor multiplicity |  |  |  | 0.241 |
| Unifocal | 35 | 21(60.0) | 14(40.0) |  |
| Multifocal | 77 | 37(48.1) | 40(51.9) |  |
| Tumor grade |  |  |  | 0.635 |
| Low | 39 | 19(48.7) | 20(51.3) |  |
| High | 73 | 39(53.4) | 34(46.6) |  |
| pT status |  |  |  | **0.003** |
| pTa/pT1 | 31 | 24(77.4) | 7(22.6) |  |
| pT2 | 34 | 16(47.1) | 18(52.9) |  |
| pT3/pT4 | 47 | 18(38.3) | 29(61.7) |  |
| pN status |  |  |  | **0.004** |
| pN- | 86 | 51(59.3) | 35(40.7) |  |
| pN+ | 26 | 7(26.9) | 19(73.1) |  |
| Abbreviations: ^a^Chi-square test; ^b^median age; ^c^median size; BC = bladder urothelial carcinoma; Significant associations are shown in bold face in the *p*-value column (*p*-value <0.05). | | | | |
