# Supplementary material for: RIPK4 promotes bladder urothelial carcinoma cell aggressiveness by upregulating VEGF-A through the NF-κB pathway
Source: Br J Cancer. 2018 Jun 5;118(12):1617–27. doi: 10.1038/s41416-018-0116-8 (PMC6008479; doi:10.1038/s41416-018-0116-8)
Supplement: Supplementary file 7 — Supplementary Table S2 [file 41416_2018_116_MOESM7_ESM.doc]

| **Supplementary Table S2.** Univariate analysis of survival in 112 cases of BC | | | |
| --- | --- | --- | --- |
| Variables | All cases | HR (95% CI) | *P* value |
| Age (years) |  |  | 0.179 |
| ≤67^a^ | 60 | 1 |  |
| ＞67 | 52 | 1.473 (0.837-2.592) |  |
| Gender |  |  | 0.203 |
| Male | 90 | 1 |  |
| Female | 22 | 1.528 (0.795-2.939) |  |
| Tumor size (cm) |  |  | 0.941 |
| ≤3.6^b^ | 58 | 1 |  |
| ＞3.6 | 54 | 1.021 (0.582-1.791) |  |
| Tumor multiplicity |  |  | 0.426 |
| Unifocal | 35 | 1 |  |
| Multifocal | 77 | 1.266 (0.708-2.265) |  |
| Tumor grade |  |  | **0.032** |
| Low | 39 | 1 |  |
| High | 73 | 2.041 (1.062-3.921) |  |
| pT status |  |  | **0.004** |
| pTa/pT1 | 31 | 1 |  |
| pT2 | 34 | 2.077 (0.838-5.150) |  |
| pT3/pT4 | 47 | 3.782 (1.648-8.677) |  |
| pN status |  |  | **＜0.001** |
| pN- | 86 | 1 |  |
| pN+ | 26 | 4.177 (2.283-7.642) |  |
| RIPK4 expressioon |  |  | **＜0.001** |
| Low expression | 54 | 1 |  |
| High expression | 58 | 4.284 (2.265-8.102) |  |
| Abbreviations: ^a^median age; ^b^median size; HR = hazard ratio; CI = confidence interval; BC = bladder urothelial carcinoma; Significant associations are shown in bold face in the *p*-value column (*p*-value <0.05). | | | |
