# Supplementary material for: RIPK4 promotes bladder urothelial carcinoma cell aggressiveness by upregulating VEGF-A through the NF-κB pathway
Source: Br J Cancer. 2018 Jun 5;118(12):1617–27. doi: 10.1038/s41416-018-0116-8 (PMC6008479; doi:10.1038/s41416-018-0116-8)
Supplement: Supplementary file 8 — Supplementary Table S3 [file 41416_2018_116_MOESM8_ESM.doc]

| **Supplementary Table S3.** Multivariate analysis of survival in 112 cases of BC | | | |
| --- | --- | --- | --- |
| Feature | HR | 95%CI | *P* value |
| Tumor grade ( low *vs.* high) | 1.934 | 0.921-4.062 | 0.082 |
| pT status ( pTa/pT1 *vs.* pT2 *vs.* pT3/pT4) | 1.481 | 0.573-3.832 | **0.036** |
| pN status ( pN- *vs.* pN+) | 2.463 | 1.299-4.672 | **0.006** |
| RIPK4 expressioon (low *vs.* high) | 3.61 | 1.774-7.346 | **＜0.001** |
| Abbreviations: HR = hazard ratio; CI = confidence interval; BC = bladder urothelial carcinoma; Significant associations are shown in bold face in the *p*-value column (*p*-value <0.05). | | | |
