# Supplementary material for: RIPK4 promotes bladder urothelial carcinoma cell aggressiveness by upregulating VEGF-A through the NF-κB pathway
Source: Br J Cancer. 2018 Jun 5;118(12):1617–27. doi: 10.1038/s41416-018-0116-8 (PMC6008479; doi:10.1038/s41416-018-0116-8)
Supplement: Supplementary file 9 — Supplementary Table S4 [file 41416_2018_116_MOESM9_ESM.doc]

| **Supplementary Table S4.** Association between the expression of RIPK4 and EMT markers in BC | | | | |
| --- | --- | --- | --- | --- |
|  |  | RIPK4 protein | |  |
| Variables | Cases | Low expression(%) | High expression(%) | *P* value^a^ |
| E-cadherin |  | 58 | 54 | **＜0.001** |
| Negative expression | 72 | 20(27.8) | 52(72.2) |  |
| Positive expression | 40 | 38(95.0) | 2(5.0) |  |
| β-catenin |  |  |  | **＜0.001** |
| Low expression | 65 | 22(33.8) | 43(66.2) |  |
| High expression | 47 | 36(76.6) | 11(23.4) |  |
| Vimentin |  |  |  | **＜0.001** |
| Negative expression | 34 | 32(94.1) | 2(5.9) |  |
| Positive expression | 78 | 26(33.3) | 52(66.7) |  |
| Fibronectin |  |  |  | **0.002** |
| Negative expression | 50 | 34(68.0) | 16(32.0) |  |
| Positive expression | 62 | 24(38.7) | 38(61.3) |  |
| Abbreviations: ^a^Fisher’s exact test; EMT = epithelial-mesenchymal transition; BC = bladder urothelial carcinoma; Significant associations are shown in bold face in the *p*-value column (*p*-value <0.05). | | | | |
