# Supplementary material for: RIPK4 promotes bladder urothelial carcinoma cell aggressiveness by upregulating VEGF-A through the NF-κB pathway
Source: Br J Cancer. 2018 Jun 5;118(12):1617–27. doi: 10.1038/s41416-018-0116-8 (PMC6008479; doi:10.1038/s41416-018-0116-8)
Supplement: Supplementary file 10 — Supplementary Table S5 [file 41416_2018_116_MOESM10_ESM.doc]

| **Supplementary Table S5.** A list of differentially expressed genes related to migration and angiogenesis of BC (Fold change > 2; *P* < 0.001) | | | |
| --- | --- | --- | --- |
| Gene | Fold change | Location | Function |
| Upregulated genes |  |  |  |
| CDH1 | 3.3 | 16q22.1 | inhibits tumor metastasis |
| CD82 | 2.71 | 11p11.2 | metastasis suppressor |
| HPSE | 3.04 | 4q21.3 | hydrolyses protein |
| MMP7 | 6.64 | 11q21-q22 | decomposes, protein hydrolysate  and promotes metastasis |
| MET | 2.51 | 7q31 | proto-oncogene, promotes cell proliferation |
| TP53 | 4.49 | 17p13.1 | induces apoptosis and cell differentiation,  inhibits proliferation |
| SMAD2 | 3.2 | 18q21.1 | cell signal transduction |
| Downregulated genes |  |  |  |
| CD44 | -4.01 | 11p13 | cell adhesion and stroma attachment |
| FN1 | -2.52 | 2q34 | participates in cell adhesion |
| FAT1 | -2.18 | 4q35 | participates in cell adhesion |
| ITGB3 | -2.43 | 17q21.32 | participates in cell adhesion |
| KISS1 | -4.27 | 1q32 | suppresses metastasis |
| MMP11 | -2.01 | 22q11.23 | decomposes, protein hydrolysate  and promotes metastasis |
| MMP13 | -4.41 | 11q22.3 | decomposes, protein hydrolysate  and promotes metastasis |
| MTSS1 | -4.27 | 8p22 | inhibits metastasis and proliferation |
| TGFB1 | -2.14 | 19q13.1 | inhibits or promotes proliferation,  promotes metastasis |
| VEGF-A | -4.24 | 6p12 | promotes proliferation, metastasis;  inhibits apoptosis |
