# Supplementary material for: RIPK4 promotes bladder urothelial carcinoma cell aggressiveness by upregulating VEGF-A through the NF-κB pathway
Source: Br J Cancer. 2018 Jun 5;118(12):1617–27. doi: 10.1038/s41416-018-0116-8 (PMC6008479; doi:10.1038/s41416-018-0116-8)
Supplement: Supplementary file 11 — Supplementary Table S6 [file 41416_2018_116_MOESM11_ESM.doc]

| **Supplementary Table S6.** Association between the expression of RIPK4 and VEGF-A, CD82, NF-κB-p65 in BC | | | | |
| --- | --- | --- | --- | --- |
|  |  | RIPK4 protein | |  |
| Variables | Cases | Low expression(%) | High expression(%) | *P* value^a^ |
| VEGF-A |  |  |  | **＜0.001** |
| Low expression | 44 | 39(88.6) | 5(11.4) |  |
| High expression | 68 | 19(27.9) | 49(72.1) |  |
| CD82 |  |  |  | 0.411 |
| Low expression | 81 | 40(49.4) | 41(50.6) |  |
| High expression | 31 | 18(58.1) | 13(41.9) |  |
| NF-κB-p65 |  |  |  | **＜0.001** |
| Negative expression | 49 | 37(75.5) | 12(24.5) |  |
| Positive expression | 63 | 21(33.3) | 42(66.7) |  |
| Abbreviations: ^a^Fisher’s exact test; BC = bladder urothelial carcinoma; Significant associations are shown in bold face in the *p*-value column (*p*-value <0.05). | | | | |
