# Supplementary material for: RIPK4 promotes bladder urothelial carcinoma cell aggressiveness by upregulating VEGF-A through the NF-κB pathway
Source: Br J Cancer. 2018 Jun 5;118(12):1617–27. doi: 10.1038/s41416-018-0116-8 (PMC6008479; doi:10.1038/s41416-018-0116-8)
Supplement: Supplementary file 12 — Supplementary materials and methods [file 41416_2018_116_MOESM12_ESM.doc]

**Supplementary materials and methods**

**Cells.** BIU87, 5637, T24, EJ, and RT4 cells were obtained from the ATCC (Rockville, MD, USA), cultured in DMEM (Invitrogen) with 10% fetal bovine serum (FBS) (GIBCO), 1 mmol/L glutamine, and 100 U/mL each of penicillin and streptomycin. The cells were cultured in a humidified incubator with 5% CO_2_ at 37 ºC. Short-tandem repeat profiling was used to authenticate the cell lines used, less than 6 months before this project was initiated, and the cells have not been in culture for more than 2 months.

**RNA isolation and quantitative real-time reverse transcription polymerase chain reaction (qRT-PCR).** Total RNA was isolated from cells and tissues using the TRIzol reagent (Life Technologies, Carlsbad, CA, USA), according to the manufacturer’s instructions. Reverse transcription using MMLV reverse transcriptase reagents (Promega, Madison, WI, USA) was performed according to the manufacturer’s protocol. qRT-PCR was carried out using GoTaq qPCR Master Mix (Promega) in a Roche LightCycler 480 II PCR system (Roche Diagnostics, Rotkreuz, Switzerland). Relative gene expression was presented as comparative threshold cycle (2-∆∆Ct) values from at least three independent experiments. β-actin was used to normalize target gene expression. The reverse transcription-PCR primer sequences were: RIPK4, forward 5′-GCCCACTACCACGTCAAGAT-3′ and reverse 5′-TTCACCATGATGTGCAGGAT-3′; VEGF-A, forward 5′-AAGGAGGAGGGCAGAATCAT-3′ and reverse 5′-ATCTGCATGGTGATGTTGGA-3′; β-actin, forward 5′-CATTAAGGAGAAGCTGTGCT-3′ and reverse 5′-GTTGAAGGTAGTTTCGTGGA-3′.

**Protein extraction and western blotting.** Total protein was extracted from freshly frozen tissue samples (tumor tissues and non-tumor control tissues) and cell lines using Radio-Iimmunoprecipitation Assay (RIPA) Lysis Buffer ((Beyotime, Shanghai, China) according to the manufacturer’s protocol. The lysates were cleared by centrifugation (12,000 rpm) at 4°C for 30 min, and protein concentrations were measured with a BCA Protein Assay Kit (Thermo Fisher Scientific, Waltham, MA, USA). Briefly, equal amounts of protein (30 μg per sample) were separated by 12% sodium dodecyl sulfatepolyacrylamide gel electrophoresis (SDS-PAGE), electro-transferred onto a polyvinylidene fluoride (PVDF) membrane (Millipore, Billerica, MA, USA) and subsequently blocked with 5% skim milk in TBST for 60 min. The membranes were incubated overnight at 4°C with antibodies against RIPK4 (Abcam; 1:500 dilution), VEGF-A (Abcam; 1:1000 dilution), NF-κB-p65 (Abcam; 1:1000 dilution), E-cadherin (Abcam; 1:1000 dilution), β-catenin (Abcam; 1:5000 dilution), Vimentin (Abcam; 1:1000 dilution), Fibronectin (Abcam; 1:500 dilution), IKK (Abcam; 1:1000 dilution), p-IKK (Abcam; 1:1000 dilution), IκB (Cell Signaling Technology; 1:1000 dilution), p-IκB (Abcam; 1:10000 dilution), RIP (Abcam; 1:1000 dilution), TRAF2 (Abcam; 1:1000 dilution), NEMO (Abcam; 1:5000 dilution), CD82 (Abcam; 1:1000 dilution), HPSE (Cell Signaling Technology; 1:500 dilution), MMP-7 (Cell Signaling Technology; 1:500 dilution), MET (Cell Signaling Technology; 1:1000 dilution), TP53 (Cell Signaling Technology; 1:500 dilution), SMAD2 (Abcam; 1:2000 dilution), CD44 (Cell Signaling Technology; 1:2000 dilution), FAT1 (Cell Signaling Technology; 1:3000 dilution), ITGB3 (Cell Signaling Technology; 1:500 dilution), KISS1 (Cell Signaling Technology; 1:500 dilution), MMP-11 (Abcam; 1:800 dilution), MMP-13 (Abcam; 1:400 dilution), MTSS1 (Abcam; 1:500 dilution), TGFB1 (Abcam; 1:200 dilution), β-actin (Aanta Cruz Biotechnology; 1:800 dilution). After three 10-min washes with TBST, the membrane was then incubated with horseradish peroxidase (HRP)-conjugated secondary antibody (Cell Signaling Technology, Danvers, MA, USA; 1:2000 dilution) for 45 min at room temperature. After washing, peroxidase activity was detected on X-ray films using an enhanced chemiluminescence detection system (ECL, Cell Signaling Technology, Danvers, MA, USA). Target protein levels were normalized with respect to β-actin protein levels.

**Immunohistochemistry (IHC).** Tissue paraffin sections were deparaffinized using dimethylbenzene and rehydrated using 100%, 95%, 90%, 80% and 70% ethanol solutions, followed by washing with phosphate-buffered saline (PBS) three times. For antigen retrieval, the slides were boiled in ethylenediamine tetraacetic acid (EDTA) buffer (pH = 8.0) for 15 min in a microwave oven. Endogenous peroxidase activity was blocked by incubation in 0.3% hydrogen peroxide at room temperature for 15 min. The slides were rinsed with PBS, and non-specific binding was prevented by incubation in 5% sheep serum albumin for 30 min. The tissue sections were then incubated with antibodies against RIPK4 (Abcam; 1:400 dilution), VEGF-A (Abcam; 1:200 dilution), p-p65 (Abcam; 1:500 dilution), CD82 (Abcam; 1:200 dilution), E-cadherin (Abcam; 1:200 dilution), β-catenin (Abcam; 1:400 dilution), Vimentin (Abcam; 1:200 dilution), and Fibronectin (Abcam; 1:400 dilution) at 4 °C overnight. The slides were washed and then were incubated for 30 min with HRP-conjugated secondary antibody (Envasion Detection kit; GK500705; Genentech, San Francisco, CA) at room temperature. After washing three times with PBS, 3, 3′-diaminobenzidine tetrahydrochloride (DAB) was used to develop the visual signal. All sections were subjected to counterstaining with hematoxylin. The total RIPK4 immunostaining score was calculated by adding the score of the proportion of positively stained tumor cells to the score for staining intensity, as determined by two pathologists who were blinded to the clinical parameters. The proportion of positively stained tumor cells was scored as follows: “0” (< 5%, negative), “1” (5–25%, sporadic), “2” (25–50%, focal) and “3” (>50%, diffuse). Staining intensity was graded using the following criteria: “0” (no staining); “1” (weak staining = light yellow), “2” (moderate staining = yellow brown) and “3” (strong staining = brown). The total immunostaining score, which ranged from 0 to 9, was calculated as the value of the proportion of positive cells score multiplied by the staining intensity score. RIPK4 abundance was defined as: ‘‘-’’ (negative, score 0), ‘‘+’’ (weakly positive, score 1-3), ‘‘++’’ (positive, score 4–6) or ‘‘+++’’ (strong positive, score 7-9). Thus, RIPK4 protein levels in BC specimens were divided into two groups: a low RIPK4 level group (RIPK4 ‘‘–’’or RIPK4‘‘+’’) and a high RIPK4 level group (RIPK4‘‘++’’ or RIPK4‘‘+++’’).

**Cell migration assay.** A chamber system comprising polycarbonate membrane inserts with 8-μm pores (Corning, Corning, NY, USA) placed in 24-well cell culture insert companion plates was used to assay cell migration. Cells (5 × 10^4^) in 200 μL DMEM containing 5% FBS were seeded in the upper chamber, with 600 μL of DMEM containing 15% FBS placed in the lower chamber, for 48 h. After incubation at 37 °C for 24 h, the cells still present in the upper chamber were removed with cotton swabs. The insert membranes were fixed with 75% methanol for 30 min, stained with 0.5% crystal violet for 60 min, and counted. Stained cells were counted in 10 random microscopic fields per membrane.

**Matrigel invasion assay.** Matrigel invasion was assayed using a chamber system comprising polycarbonate membrane inserts with 8-μm pores (Corning) placed in 24-well cell culture insert companion plates. The inserts were coated with a thin layer of 0.5 mg/ml Matrigel Basement Membrane Matrix (BD Biosciences, Bedford, MA, USA). Briefly, transfected cells were resuspended in DMEM containing 5% FBS. Cells (4 × 10^5^) in 200 μL of growth medium were placed in the upper chamber, and 600 μL of growth medium containing 15% FBS filled the lower chamber. The cells were incubated at 37 °C for 48 h, after which, non-migratory cells were removed from the upper chamber using a cotton swab. Cells that had invaded the bottom of the filter were fixed with 75% methanol for 30 min, stained with 0.5% crystal violet for 60 min, and counted. Stained cells were counted in 10 random microscopic fields per membrane.

**Human umbilical vein endothelial cells (HUVECs) tube formation assay.** The HUVECs tube formation assay was performed by first pipetting 200 μL of Matrigel (BD Biosciences) into wells of a 24-well plate, and then polymerizing for 30 min at 37 °C. HUVECs (2×10^4^ cells in 200 μL of conditioned medium) were then added to the wells and incubated at 37 °C, in 5% CO_2_ for 12 h. A bright-field microscope at 100× magnification was used to capture the images. Measuring the total length of the complete tubule structures quantified the capillary tubes.

**Immunoflourescence staining.** Cells grew on glass coverslips in 6-well plate. After cells grew to cover the glass coverslips, the cells were fixed with 4% paraformaldehyde for 30 min. The cells were washed with PBS three times and then treated with 1% Triton-x-100 for 10 min to enhance penetration. The cells were pretreated with 10% normal goat serum for 30 min, and covered with anti-NF-κB-p65 (Abcam; 1:300 dilution) and anti-E-cadherin (Abcam; 1:200 dilution), β-catenin (Abcam; 1:400 dilution), Vimentin (Abcam; 1:200 dilution), and Fibronectin (Abcam; 1:400 dilution) at 4 °C degree overnight. The cells were washed with PBS and incubated with a secondary antibody (FITC-labeled rabbit anti-mouse lgG) for 40 min at room temperature. Immunofluorescence images were recorded using a LeicaTCS-SP5 confocal laser scanning microscope (Heidelberg, Germany.)

**Luciferase activity assays.** Cells (2×10^4^) were seeded in 48-well plates in triplicate and allowed to settle for 24 hours. One hundred nanograms of the NF-κB luciferase reporter plasmid or the control-luciferase plasmid, plus 1 ng of the pRL-TK Renilla plasmid (Promega), were transfected into the cells using Lipofectamine 2000 according to the manufacturer's instructions. A Dual Luciferase Reporter Assay Kit (Promega) was used to measure the Luciferase and Renilla signals 24 h post-transfection, according to the manufacturer's instructions.

**Electrophoretic mobility shift assay (EMSA).** A LightShift Chemiluminescent EMSA Kit from Pierce Biotechnology was used to perform the EMSA tests, according to the manufacturer's standard protocol. The EMSA DNA probes comprised: NF-κB: sense, 5′-AGTTGAGGGGACTTTCCCAGGC-3′, antisense, 5′-GCCTGGGAAAGTCCCCTCAAC-3′; OCT-1: sense, 5′-TGTCGAATGCAAATCACTAGAA-3′, antisense, 5′-TTCTAGTGATTTGCATTCGACA-3′.

**Lung metastasis model.** BALB/c-nu mice (5–6 weeks old, 18–20 g) were obtained from the Experimental Animal Center, Central South University (Changsha, China). The Institutional Animal Care and Use Committee of Central South University approved all the experimental procedures. The BALB/c-nude mice were divided randomly into six groups (n=7/group). BC cells (2.5 × 10^6^) were injected intravenously through tail vein on day 0. On day 60, the mice were sacrificed. Their lungs were dissected and fixed in 10% buffered formalin to count the metastatic nodules. The number of metastatic nodules on the surface of each set of lungs was counted by visual inspection under a stereoscopic dissecting microscope.
